# The influence of action on perception spans different effectors

**DOI:** 10.3389/fnsys.2023.1145643

**Published:** 2023-05-02

**Authors:** Annalisa Bosco, Pablo Sanz Diez, Matteo Filippini, Patrizia Fattori

**Affiliations:** ^1^Department of Biomedical and Neuromotor Sciences, University of Bologna, Bologna, Italy; ^2^Alma Mater Research Institute for Human-Centered Artificial Intelligence (Alma Human AI), University of Bologna, Bologna, Italy; ^3^Carl Zeiss Vision International GmbH, Aalen, Germany; ^4^Institute for Ophthalmic Research, Eberhard Karls University Tüebingen, Tüebingen, Germany

**Keywords:** object properties, reaching, grasping, eye movements, walking

## Abstract

Perception and action are fundamental processes that characterize our life and our possibility to modify the world around us. Several pieces of evidence have shown an intimate and reciprocal interaction between perception and action, leading us to believe that these processes rely on a common set of representations. The present review focuses on one particular aspect of this interaction: the influence of action on perception from a motor effector perspective during two phases, action planning and the phase following execution of the action. The movements performed by eyes, hands, and legs have a different impact on object and space perception; studies that use different approaches and paradigms have formed an interesting general picture that demonstrates the existence of an action effect on perception, before as well as after its execution. Although the mechanisms of this effect are still being debated, different studies have demonstrated that most of the time this effect pragmatically shapes and primes perception of relevant features of the object or environment which calls for action; at other times it improves our perception through motor experience and learning. Finally, a future perspective is provided, in which we suggest that these mechanisms can be exploited to increase trust in artificial intelligence systems that are able to interact with humans.

## Introduction

At the basis of a successful behavior there is the interplay between perception and action. Typically, perception informs the action mechanism regarding the features of the environment and this mechanism is responsible for changes in the environment. If, on the one hand, it is doubtless that perception influences the action, the influence of action on perception cannot be taken for granted to the same extent. Starting from such a consideration, this review aims to examine the influence of action on visual perception of different properties of objects (i.e., size, orientation, and location) focusing on the actions performed by different motor effectors such as the eye, the hand, and the leg. Since two phases can be distinguished when looking at the influence of action on perception, namely planning and execution, in the following sections we provide a separate overview of some of the studies that explore the effect of action planning on the perception of the object/stimulus, and of those that examine action execution and its effect on perception.

## The effect of action planning on perception

As perceivers, we receive, on a daily basis, a wide variety of information concerning the features of the surrounding environment. As active players, we constantly explore this environment based on the sensory processing of those stimuli related to our goals/intentions and subsequent actions. For example, everyday tasks such as grasping a cup or the handle of a frying pan are highly precise actions that we perform automatically; however, these involve a complex sensorimotor approach, many aspects of which are still unknown.

An action, an intended and targeted movement, is distinguished by several sequential sections that organize its processing and work in close coordination with perception (Hommel et al., 2016). Within this processing (assessment of environmental information, location in three-dimensional space, and selection, integration, and initiation of the action), action planning represents a fundamental component (Hommel et al., 2016; Mattar and Lengyel, 2022).

Action planning is specified as a process which considers the execution of actions based on the environment and expected outcomes (Sutton and Barto, 1998; Mattar and Lengyel, 2022). Action planning could be referred to as the period between the decision phase and the initial impulse phase. During the action planning phase, the player generates an action goal (based on the temporal and spatial properties of the environment) which is then transferred to the motor system to achieve that specific purpose. That is, first the information is organized and subsequently integrated into a plan. This particularity provides plasticity and favors the adaptation to possible changes to the input information and goals (Mattar and Lengyel, 2022). For example, when grasping an object, it can be observed how the hand adjusts to the intrinsic properties of that object (Jeannerod, 1981), which hints at the relevance of action planning in the interaction between the environment and the final goal. In fact, input information is processed in parallel by pathways acting in a shared action-perception framework (Prinz, 1990; Hommel et al., 2001), within which planning itself has been observed to influence (Hommel et al., 2001, 2016; Witt, 2018).

Notwithstanding the considerable scientific literature on how planning contributes to cognitive processes, the current findings merely give us a glimpse of the long road ahead. Here, in the following sections, we outline the most important behavioral studies regarding the impact of action planning on perception.

### The eye domain

Our visual system captures primordial information which guides our actions. Once the visual environment and objects of interest are defined, the visuo-spatial information is then transferred in order to plan, execute, and control those goal-directed actions (Hayhoe, 2017). The impact of vision on motor actions has always been a topic of great scientific interest (Prablanc et al., 1979; Desmurget et al., 1998; Land, 2006, 2009). Several decades ago, groundbreaking studies were already describing how vision improves goal-directed movement accuracy (Woodworth, 1899; Bernstein, 1967). Since then, subsequent studies have sought to investigate how vision influences planning, execution, and control of movements.

Visual information greatly contributes to the action planning phase. During planning, the presence of visual feedback regarding the limb is paramount. For example, motor actions are more accurate when visual feedback is provided during action planning, regardless of whether the limb is visible or not during the action (Prablanc et al., 1979; Conti and Beaubaton, 1980; Pelisson et al., 1986; Velay and Beaubaton, 1986; Elliott et al., 1991, 2014; Rossetti et al., 1994; Desmurget et al., 1995, 1997; Coello and Grealy, 1997; Bagesteiro et al., 2006; Bourdin et al., 2006; Sarlegna and Sainburg, 2009).

Indeed, vision plays a key role in action planning since movements are apparently planned as vectors based on the extrinsic coordinates of the visual environment (Morasso, 1981; Flanagan and Rao, 1995; Wolpert et al., 1995; Sarlegna and Sainburg, 2009). Once visual information has been extracted, planning should consider those properties that will shape further actions. An example may be that of driving a car and approaching an intersection. Our visual system extracts information regarding the location and movement of other cars, pedestrians, and traffic signals at the intersection. Based on the extrinsic coordinates of the visual environment, during action planning we determine the appropriate vectors for our movements, such as accelerating, braking, or turning, that allow us to navigate the intersection safely and efficiently.

In a common framework, two stages within action planning have been suggested: the primary stage, in which vision is fundamental to determine the visuo-spatial attributes (target, limbs, and environment), and the secondary stage, in which the primary input is transformed into motor commands to generate the action (Sarlegna and Sainburg, 2009). Therefore, in a normal context, during action planning, vision provides the relevant information which facilitates the success of an action.

Acquiring visuo-spatial information would not be possible without eye movements. If a target falls in the peripheral visual field, eye movements assist in conveying the exact location of the target. Suppose we want to reach for an object. Considering the retinal spatial resolution, when a target of interest to be reached is identified, the region of highest retinal resolution should be focused on that target (Liversedge and Findlay, 2000; Land, 2006). To this end, before the reaching action begins, the eyes perform a saccadic movement toward the object and then fixate it constantly until it is reached by the hand. Within this brief scenario, the relevance of eye movements, which continuously support the coupling of vision and action, can be appreciated (Land, 2006, 2009; Hayhoe, 2017; de Brouwer et al., 2021).

Research into the interaction between the visual and motor systems has shown how the eyes constantly support and guide our actions in multiple dynamic tasks (Angel et al., 1970; Biguer et al., 1982; Pelisson et al., 1986; Land, 1992; Land et al., 1999; Neggers and Bekkering, 1999, 2000, 2001, 2002; Johansson et al., 2001; Patla and Vickers, 2003). For example, Land et al. (1999) demonstrated that eye movements are directed to those objects involved in our daily actions. In Neggers and Bekkering (1999, 2000, 2001, 2002) studies, a mechanism of gaze anchoring during hand actions was elegantly demonstrated. They observed that during reaching movements observers did not make saccadic movements toward another target until the hand had arrived at the target of interest. Similar findings were reported by Johansson et al. (2001). They instructed participants to reach and grasp a bar which they subsequently had to move while avoiding obstacles and finally attach to a switch. They reported that gaze fixation was focused on those points that were critical to the action. That is, eye movements continuously guided the action to grasp, navigate, and attach the object (Johansson et al., 2001). In other studies, it was shown that fixation patterns differ when an object is grasped or viewed passively (Vishwanath and Kowler, 2003; Brouwer et al., 2009). Both studies showed that during visualization, fixation patterns were focused on the object's center of gravity, whereas during grasping, fixation was affected by the contact zone of the index and thumb digits. Interestingly, Brouwer et al. (2009) observed that saccadic reaction times were slower in the grasping task as compared to the visualization task. This outcome reflects that the onset of eye movement was dependent on action planning, i.e., in those conditions in which the eye and hand participated in the same process.

**Figure 1:**
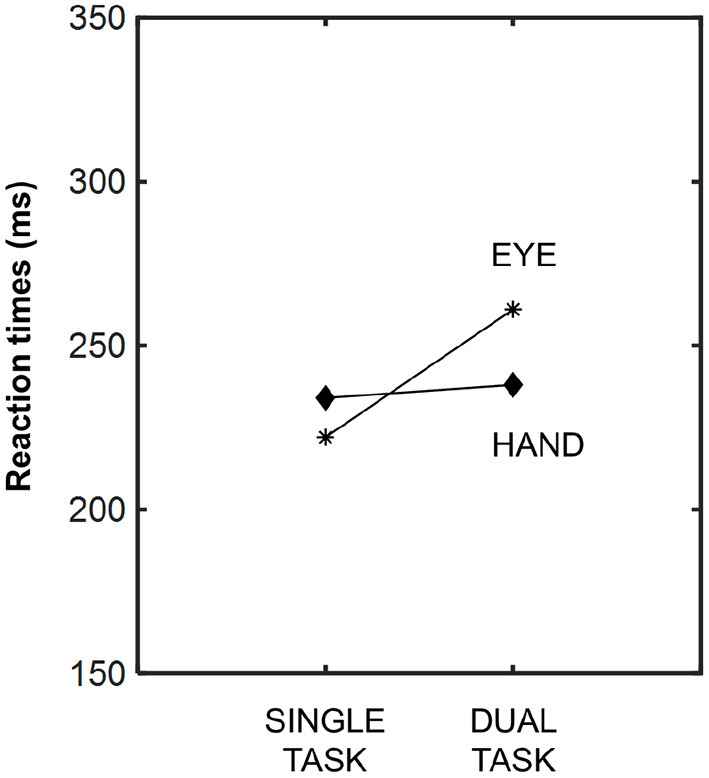
Averaged latencies of eye and hand responses, measured in milliseconds, under both experimental conditions: single- and dual-task. Modified from Bekkering et al. (1994).

The eye reaction time latencies relative to the action have already been reported in several studies (Bekkering et al., 1994, 1995; Lünenburger et al., 2000; Pelz et al., 2001; Hayhoe et al., 2003). Bekkering et al. (1994) measured eye and hand motor response latencies using single- and dual-task methodologies. Like Brouwer et al. (2009), and as can be appreciated in Figure 1, saccade reaction time latencies were highest in the dual approach, i.e., when both the eye and hand simultaneously moved toward the visual target. Hand latencies were similar in both the single and dual tasks (Bekkering et al., 1994). Conversely, in another study, lower saccadic reaction time latencies were reported when the eye and hand moved simultaneously toward a common target (Lünenburger et al., 2000). Perhaps the type of planned action (pointing, reaching, grasping, etc.) is decisive within this interference effect. Longer processing times may be required according to the type of action planned, and, thus, eye reaction times could be affected differently (Brouwer et al., 2009). These findings demonstrate that these motor systems (eye-limb) are not independent from each other, and that they share synergistic processes when targeted to the same goal.

Recent studies have revealed how eye movements support selection and action planning toward a goal. Particularly, exploration of the eye-limb relationship in naturalistic tasks has revealed how eye movements provide continuous information from the visual environment, generating a context of intrinsic properties and spatial coordinates during action planning to effectively guide future movements (Zelinsky et al., 1997; Land et al., 1999; Pelz et al., 2001; Brouwer et al., 2009). In tasks involving jar-opening or hand-washing it has been observed that reaching actions are preceded by anticipatory fixations toward the target of interest (Pelz et al., 2001; Hayhoe et al., 2003). These fixations occur during action planning and help the observer to obtain decisive spatial information to assist in the future action (Hayhoe et al., 2003). Other activities, such as walking or driving over difficult and tortuous surfaces, have shown how visuo-spatial information derived from eye movements is primordial during action planning (Land and Lee, 1994; Patla and Vickers, 2003; Land, 2006). For example, while walking, gaze fixation anticipates action by 0.8–1.1 s on average (Patla and Vickers, 2003; Land, 2006). This suggests that during planning, the visual system acts as an anticipatory system, in a feedforward manner, for the execution of the action.

Although previous studies focused on the role of the eyes as support in the planning of actions performed by other motor effectors, multiple studies have extensively examined the impact of eye motor planning on visual perception within the oculomotor system. These investigations have shown that spatial perception is enhanced at the location where the eye movement is intended to go, shortly before its execution (Hoffman and Subramaniam, 1995; Deubel and Schneider, 1996; Neggers et al., 2007). For example, research has shown that saccade target selection is influenced by object recognition (Deubel and Schneider, 1996), and that visual attention can influence the planning and execution of saccadic eye movements (Hoffman and Subramaniam, 1995). Additionally, a coupling between visuospatial attention and eye movements has been observed (Neggers et al., 2007), with attention often following the gaze. This coupling can be disrupted when transcranial magnetic stimulation is applied to the frontal eye fields, suggesting a causal relationship between attention and eye movements (Neggers et al., 2007). These outcomes may imply that the process of eye motor planning can have a significant impact on perception. Although the exact mechanisms underpinning this impact are not yet fully known, it is believed that the coordinated activity of multiple brain regions and systems, including the saccadic system, vestibular system, and attentional processes, is at play.

### The hand domain

Over the past few decades, the scientific literature has provided compelling evidence as to how perception is biased by the planning of arm movements, such as reaching and grasping (Musseler and Hommel, 1997; Prinz, 1997; Craighero et al., 1999; Wohlschläger, 2000; Hommel et al., 2001; Knoblich and Flach, 2001; Wühr and Müsseler, 2001; Hamilton et al., 2004; Kunde and Wuhr, 2004; Fagioli et al., 2007; Wykowska et al., 2009, 2011; Kirsch et al., 2012; Kirsch and Kunde, 2013; Kirsch, 2015). From the perceiver's point of view, it is intriguing to consider the fact that when planning a reaching or grasping movement toward an object, the perception of the object is somehow influenced. For example, when reaching for a cup of coffee, the perceiver's visual system considers the cup's location and orientation relative to the perceiver's body. The perceived properties of the cup may also be influenced by the planned action, as the perceiver's motor system may need to make adjustments based on these properties in order to successfully grasp the cup. This suggests that the motor system is not only involved in executing actions, but also in shaping perception based on the perceiver's intended actions. In fact, multiple perceptual aspects, such as orientation, size, luminance, location, motion, among many others, have been reported as target features that are directly influenced by action planning (Musseler and Hommel, 1997; Craighero et al., 1999; Wohlschläger, 2000; Zwickel et al., 2007; Lindemann and Bekkering, 2009; Kirsch et al., 2012). For example, studies by Kirsch have shown how planning itself interferes with distance perception and, therefore, with target spatial location (Kirsch et al., 2012; Kirsch and Kunde, 2013; Kirsch, 2015).

This action(planning)-perception interaction is dependent on whether the goal is related or not to the action. When there is a direct relationship between goal and action, perception is facilitated by the planning of the action, whereas when the two are independent, action planning interferes with perception (Hommel et al., 2016).

Several benchmark studies carried out in the 1990s and 2000s demonstrated various scenarios exhibiting facilitation and interference. Based on a set of five experiments, Musseler and Hommel (1997) reported the impact of action planning on the direction perception of a visual stimulus. Direction perception (right or left) was influenced by action planning concurrence (right or left button press). Specifically, identifying the direction of a right-pointing stimulus was more costly after planning a right button press (Musseler and Hommel, 1997). Given the common code (Hommel et al., 2001), action planning toward a concrete direction led to an interference scenario, i.e., the share-code weighting favored action over perception (Musseler and Hommel, 1997; Hommel et al., 2016).

Subsequent studies corroborated the interaction between perception and action planning processes. In Wohlschläger (2000) study, observers had to report the perceived motion direction of projected discs while turning a knob in a designated direction. Hand motion direction biased subjects' motion perception. Under a similar experimental approach to that of Craighero et al. (1999), Lindemann and Bekkering (2009) instructed volunteers to reach, grasp, and subsequently rotate an x-shaped manipulandum following the visual go signal's onset. Here, a tilted bar (-45° or +45°) served as the visual go signal. Volunteers detected the onset of the go signal faster in the congruent conditions, in which the go signal, and action planning presented the same direction (Lindemann and Bekkering, 2009). These findings imply that perception was facilitated in the direction in which the action had been previously planned. In contrast, like Musseler and Hommel (1997), Zwickel et al. (2007) reported action (planning)-perception coupling but in an interference scenario. In their study, reaction times were longer when movement deviations agreed with the action planning direction (Zwickel et al., 2007). Interference situations have also been reported by other authors (Schubö et al., 2001; Hamilton et al., 2004; Zwickel et al., 2010), indicating that the action (planning)-perception coupling is dependent on whether the perceived target is linked or not to the planned action.

**Figure 2:**
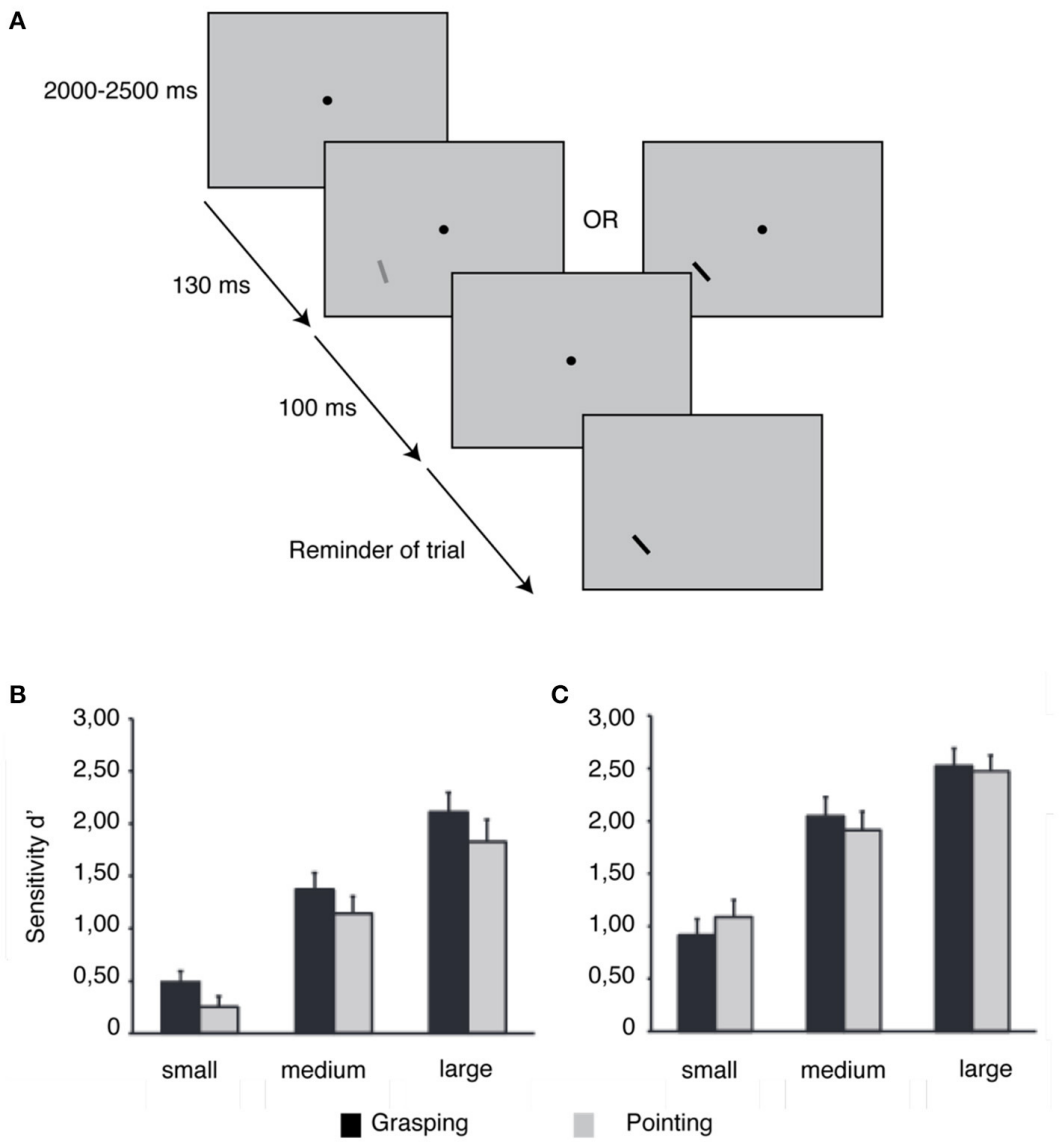
Effect of grasping and pointing planning on orientation and luminance detection. **(A)** Experimental approach. Experiments 1 and 2 used a similar stimulus display, which included a fixation spot and two bars. Participants were instructed to execute an action after a go-cue signaled by the appearance of the first bar. The second bar was either rotated slightly (Experiment 1) or differed in luminance (Experiment 2) from the first bar. **(B)** In Experiment 1, participants showed better orientation discrimination when planning a grasping action rather than when planning a pointing action. **(C)** Experiment 2 did not reveal any consistent change in luminance discrimination between grasping and pointing planning. Modified from Gutteling et al. (2011).

Recent research has proven the relevance of the type of action planning in how perception is biased (Bekkering and Neggers, 2002; Fagioli et al., 2007; Symes et al., 2008; Wykowska et al., 2009, 2011; Gutteling et al., 2011). Bekkering and Neggers (2002) instructed observers to point at or grasp an object with a specific orientation and color. The authors found that while color errors were identical in both approaches, the number of orientation errors was lower in the grasping scenario (Bekkering and Neggers, 2002). Gutteling et al. (2011) asked participants to perform a grasping or pointing movement simultaneously with an orientation or luminance discrimination task (see Figure 2). Orientation sensitivity increased when planning a grasping action, as opposed to a pointing action. Size, location, and luminance have also been described as being perceptually dependent attributes of the type of action planning (Fagioli et al., 2007; Wykowska et al., 2009, 2011; Kirsch et al., 2012; Wykowska and Schubö, 2012; Kirsch and Kunde, 2013). Fagioli et al. (2007) revealed that planning a grasping action improved the ability to detect deviations in object size, while planning a reaching action facilitated the detection of location deviations. Studies by Wykowska et al. (2009) and Wykowska and Schubö (2012) corroborated the finding that planning to grasp improves size perception, while planning to reach enhances luminance perception.

All the above-mentioned scientific evidence seems to support the common coupling of action(planning)-perception. Planning an action primes those perceptual dimensions that can enhance one's own action (Hommel et al., 2001; Wykowska et al., 2009).

**Figure 3:**
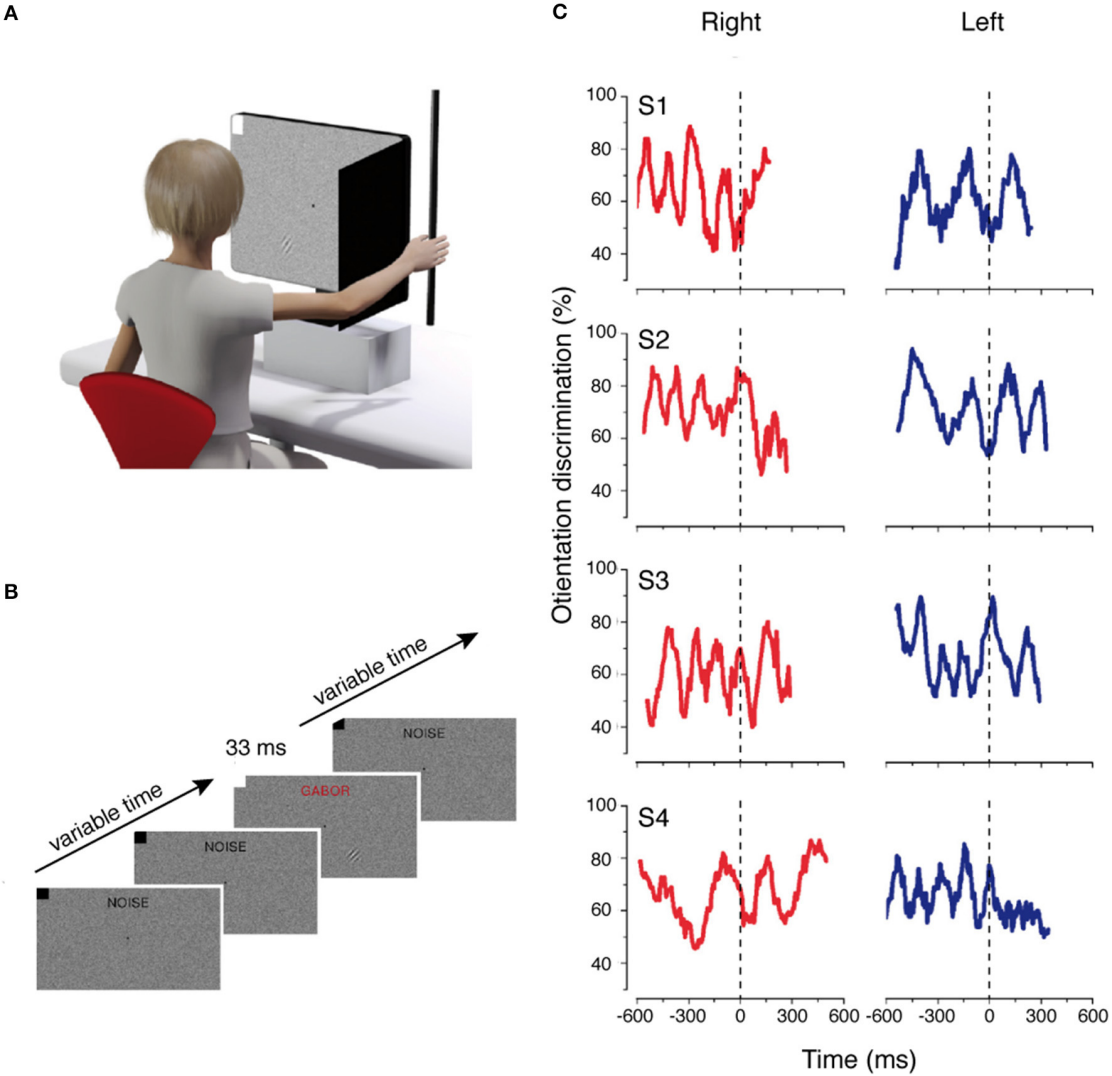
Rhythmic oscillations of contrast sensitivity synchronized with hand movements. **(A)** Experimental setup of the motor and visual tasks. **(B)** Example of trial sequence. Visual noise and fixation point were presented from the beginning of the trial to the end. At a random time from the start of the trial, a Gabor stimulus was displayed to the lower right or to the lower left of fixation. **(C)** Time course of the orientation discrimination responses for each participant aligned with the onset of the hand movement. Modified from Tomassini et al. (2015).

The majority of studies cited have shown that the motor system dynamically modulates the incoming perceptual information. However, these modulations have been observed when the perceptual information is intermixed with attentional and decisional mechanisms because they are strictly related to the motor response (i.e., Gutteling et al., 2011). Relevant literature was dedicated to understanding the temporal tuning of incoming perceptual information at very early cortical stages. To do this, different studies measured the contrast sensitivity of a brief visual stimulus that was not correlated with the action to be performed, and that was presented at different times during motor planning and execution. These studies used contrast sensitivity because it represents and reflects the activity of the primary visual cortex, since it has been demonstrated that the change of contrast visibility requires a modulation at this early cortical level (Boynton et al., 1999). Furthermore, it has recently been demonstrated that both sensory and motor processes are regulated by a rhythmic process that reflects the oscillations of neuronal excitability (Buzsáki and Draguhn, 2004; Thut et al., 2012). Combining all these pieces of evidence, Tomassini et al. (2015) evaluated whether the rhythmic oscillations of visual contrast sensitivity were also present when synchronizing the perceptual information with the onset of a reaching and grasping movement. They found that the oscillations in contrast sensitivity emerged around 500 ms before movement onset, during action planning, even if perception was not related to the motor task (see Figure 3). These findings were extended in an electroencephalographic (EEG) study, in which the same group demonstrated that motor planning is combined with perceptual neural oscillations (Tomassini et al., 2017). The perceptual “action-locked” oscillations were also observed when the movements were performed with the eyes (Benedetto and Morrone, 2017; Benedetto et al., 2020). In this study, the results showed that saccadic preparation and visual contrast sensitivity oscillations are coupled, suggesting a functional alignment of the saccade onset with the visual suppression (Benedetto and Morrone, 2017).

### The leg domain

The above-discussed research focuses on peripersonal space. However, it has been observed that the impact of action planning on perception can extend to the leg effector domain, resulting in facilitation effects on the perception of extrapersonal space. Several studies have shown that, when viewing objects in our extrapersonal space, we scale the perceived distance according to our intended motor action. For example, if we plan to walk a certain distance, we perceive the distance based on the amount of walking effort needed to traverse it, while, if we intend to throw a ball, the perceived distance is based on the amount of throwing effort required (Witt et al., 2004; Proffitt, 2006; Witt and Proffitt, 2008). The way we perceive our environment seems to be influenced by the specific actions we anticipate taking, with perception being adjusted based on an optimal cost-benefit principle (Proffitt, 2006). Recently, Fini et al. (2014, 2015a,b) used a virtual paradigm to investigate the influence of anticipated actions on spatial perception. Participants were asked to judge the location of an object positioned at progressively increasing or decreasing distances from a reference frame. They noticed that participants perceived the target object to be closer to their own body when they intended to move toward it compared to when they had no intention of moving. This effect was not observed when the target object was compared to another static object (Fini et al., 2015a). Additionally, studies have demonstrated that when leg actions such as walking or running are primed, the portion of extrapersonal space judged as near in other-based coordinates is significantly expanded (Fini et al., 2017), together with an extension of peripersonal space during full-body actions such as walking compared to standing (Noel et al., 2015). These findings suggest that visual perception of the physical environment beyond our body is heavily influenced by our actions, intentions, and physical abilities. Apparently, the main way of exploring the extended environment seems to be through locomotion, as it is the only way to cover distances and access information from more distant locations in the extrapersonal space compared to near extrapersonal locations, where information can be extracted from different sources (di Marco et al., 2019).

## The effect of action execution on perception

Human ability to perform actions impacts the visual perception of objects/targets. This represents the framework within which the influence of action execution on perception is typically explained. The action-specific effects indicate all the effects generated from the ability to act on spatial perception (Proffitt, 2006, 2008). The first study suggesting that spatial perception was influenced by the ability to perform an action was carried out by Bhalla and Proffitt (1999). They showed that the perception of hill slant was influenced by the physiological potential. In fact, if the energetic costs required to climb them increased, the hills were estimated to be steeper. Following this work, several researchers have focused and expanded this concept beyond the physiological potential; however, these studies focus on other aspects of action. For example, softball players who were good at hitting the ball estimated it as being bigger compared to others (Witt and Proffitt, 2005; Gray, 2013). Similarly, archers who had a better shot than others estimated the target as bigger (Lee et al., 2012). Parkour athletes judged walls as lower compared to non-parkour athletes (Taylor et al., 2011), and good tennis players judged the net as being lower (Witt and Sugovic, 2010).

Another study examining a different branch of action-specific effects analyzed the affordance of the object, in other words, the possibility to act on or with an object (Gibson, 1979). Typically, the measurement of affordance perception is carried out by assessing the point at which an action is perceived as barely possible. For example, some studies have explored the width of a doorway that is perceived as being just possible to pass through, or the height of a step at which the affordance of stepping up is perceived (Warren, 1984; Mark, 1987; Warren and Whang, 1987). Other examples are studies in which people with broader shoulders perceived doorways to be smaller compared to people with narrower shoulders (Stefanucci and Geuss, 2009), or studies in which a target is presented beyond the distance of the arm's reach: the target is perceived as being closer when the participants use a reach-extending tool to reach the target and more distant when they reach without the tool (Witt et al., 2005; Witt and Proffitt, 2008; Witt, 2011; Davoli et al., 2012; Osiurak et al., 2012; Morgado et al., 2013). Given all these remarkable data, the next section focuses on the action-specific effects on perception as a function of the specific effector used, expanding the panorama to other investigation modes.

### The eye domain

In the eye realm, the effect of saccade execution on perception has been investigated through saccadic adaptation and perisaccadic mislocalization mechanisms. Saccadic adaptation allows researchers to study how saccade amplitudes change according to changes in the post-saccadic target shift. This change can be either parallel or orthogonal to the main direction of the saccade. In other words, it is well established that saccade amplitudes adapt when a small target is horizontally shifted during saccade execution to another position in relation to the initial one (McLaughlin, 1967; Miller et al., 1981; Deubel, 1987; Watanabe et al., 2003; Hopp and Fuchs, 2004; Kojima et al., 2005; Ethier et al., 2008; Rahmouni and Madelain, 2019). Further studies investigated the possibility of the saccadic system sharing common coordinates with other domains. In fact, several researchers have demonstrated that the modification of motor variables induced by saccade adaptation leads to a concomitant modification of the perceived location of the target when the localization is executed by a pointing movement or by a perceptual report (Bahcall and Kowler, 1999; Awater et al., 2005; Bruno and Morrone, 2007; Collins et al., 2007; Zimmermann and Lappe, 2010; Garaas and Pomplun, 2011; Gremmler et al., 2014).

**Figure 4:**
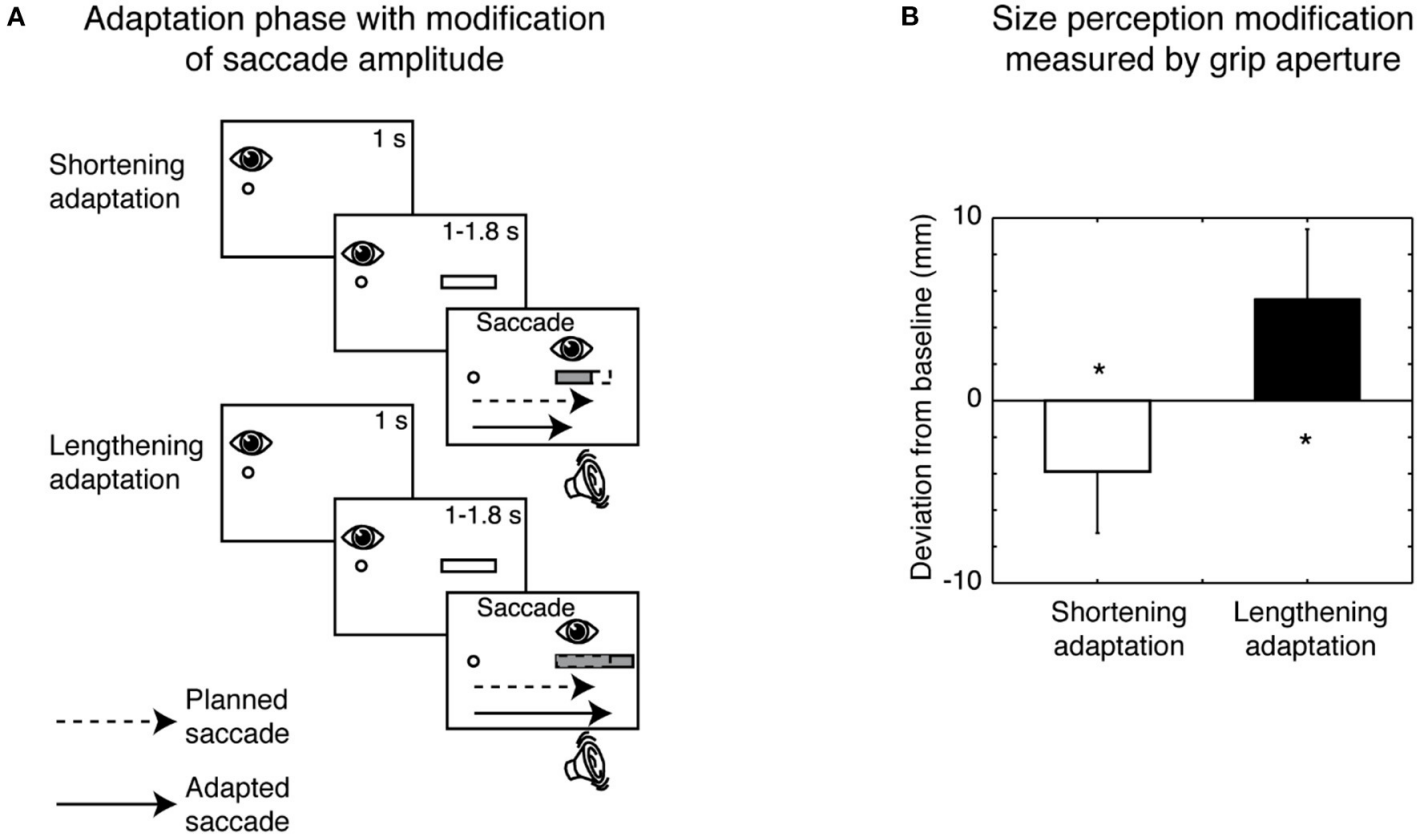
Size perception modification induced by saccadic adaptation. **(A)** Top row, Shortening adaptation condition. The fixation point was presented at the start of the trial. After 1 s, a bar appeared, but participants had to continue to focus on the fixation target. After a randomized time, an acoustic signal indicated the possibility of executing a saccade toward the bar. Then the bar was decreased in size by 30% of its length at the right border as soon as the saccade was detected. Bottom row, Lengthening adaptation phase. This condition was identical to the shortening adaptation condition, with the only difference being that the bar was increased in size by 30% during saccade execution. **(B)** Mean deviation of grip aperture from baseline in shortening adaptation (white column) and lengthening (black column) adaptation. The data were averaged across subjects and sizes. Error bars indicate SE. *p < 0.05, significant deviations from baseline (modified from Bosco et al., 2015).

A particular application of the saccadic adaptation paradigm was developed using spatially extended targets that, during the saccade, systematically changed their horizontal size (Bosco et al., 2015), and in reading studies (McConkie et al., 1989; Lavergne et al., 2010). In particular, the manipulation used in Bosco et al. (2015) influenced the target visual perception. The modification of size perception occurred according to the direction of saccadic amplitude adaptation: if the saccade was adapted to a smaller amplitude, target size was perceived as being smaller; if the saccade adapted to a larger amplitude, target size was perceived as being larger (Bosco et al., 2015). The scheme of the adaptation phase paradigm and the consequent size perception modification measured by grip aperture of the hand is shown in Figure 4.

**Figure 5:**
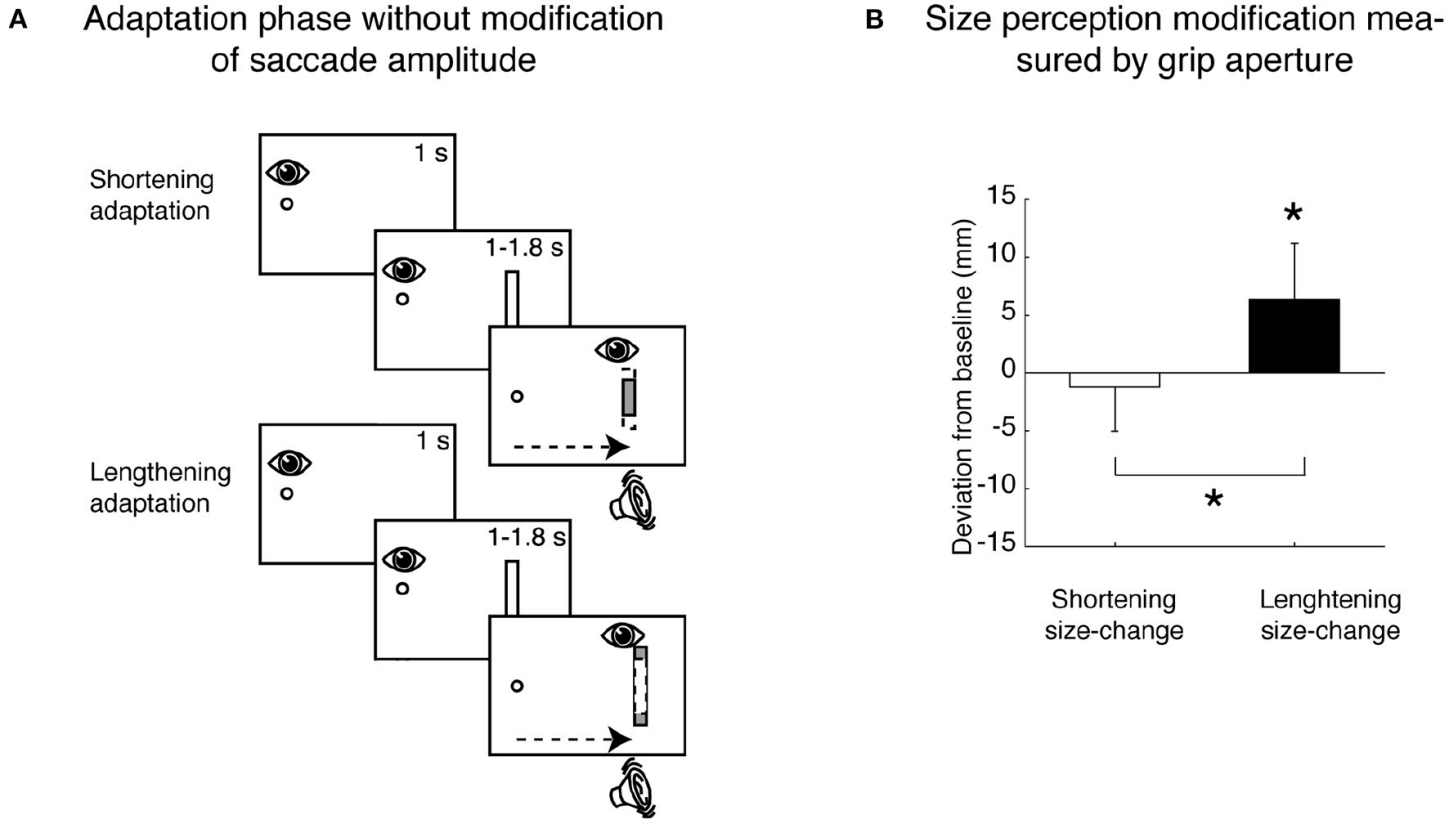
Size perception modification not induced by saccadic adaptation. **(A)** Top row, Shortening adaptation condition. The fixation point was presented at the start of the trial. After 1 s, a bar appeared, but participants had to continue to focus on the fixation target. After a randomized time, an acoustic signal indicated the possibility of executing a saccade toward the bar. Then the bar was symmetrically decreased in size by 30% of its length as soon as the saccade was detected. Bottom row, Lengthening adaptation. This was identical to the shortening adaptation, with the only difference being that the bar was symmetrically increased in size by 30% during the execution of the saccade. **(B)** Mean deviation of size perception (grip aperture) from baseline for the shortening (white column) and the lengthening (black column) adaptation trials. The data were averaged across participants and sizes. Details as in Figure 4 (modified from Bosco et al., 2020). ^*^p < 0.05, significance level.

However, recent studies have shown that change in perception of visual features is present also without saccadic adaptation (Herwig and Schneider, 2014; Herwig et al., 2015, 2018; Valsecchi and Gegenfurtner, 2016; Paeye et al., 2018; Köller et al., 2020; Valsecchi et al., 2020). This phenomenon occurs with the following features: the perception of spatial frequency (Herwig and Schneider, 2014; Herwig et al., 2018), shape (Herwig et al., 2015; Paeye et al., 2018; Köller et al., 2020), and size (Valsecchi and Gegenfurtner, 2016; Bosco et al., 2020; Valsecchi et al., 2020). For example, Bosco et al. (2020) used a manipulation consisting in the systematic shortening and lengthening of a vertical bar during a horizontal saccade aimed to do not modify the saccade amplitude; by these conditions, they observed a significant difference in perceived size after the saccade execution (see Figure 5A for the scheme of saccadic adaptation paradigm in Bosco et al., 2020). This finding suggested that the modification of size perception does not rely on the modified saccadic amplitude induced by saccadic adaptation mechanisms (see Figure 5B, Bosco et al., 2020). In the study by Valsecchi et al. (2020), it was shown that saccadic adaptation and size recalibration share the same temporal development. However, size recalibration of the visual stimuli was also present in the opposite hemifield, but saccadic adaptation did not suggest that distinct mechanisms were involved. Although the modification of saccadic parameter induced by saccadic adaptation is not the causal mechanism for the modification of stimulus property perception, the shift of the target image from the periphery to the fovea, typically performed by a saccade, remains the potential cause of the observed object perception modification.

A considerable body of literature has shown that the visual stimuli briefly presented just before the onset of a saccade, or during it, are mislocalized and perceived as being closer to the saccade target (Matin and Pearce, 1965; Honda, 1989; Schlag and Schlag-Rey, 1995; Ross et al., 1997). In other terms, this mislocalization consists in a shift of apparent position in the direction of the saccade (Honda, 1989, 1995; Schlag and Schlag-Rey, 1995; Cai et al., 1997; Lappe et al., 2000) and a compression of positions onto the target location of the saccade (Bischof and Kramer, 1968; Ross et al., 1997; Lappe et al., 2000). The shift is attributed to a mismatch between the actual eye position during the saccades and the predicted position originating from an internal corollary discharge (Duhamel et al., 1992; Nakamura and Colby, 2002; Kusunoki and Goldberg, 2003; Morrone et al., 2005).

Interestingly, the compression effect is primarily observed parallel to the saccade direction (Ross et al., 1997), and also in the orthogonal direction (Kaiser and Lappe, 2004; Zimmermann et al., 2014, 2015), suggesting that a linear translation of the internal coordinate system is a reductive explanation. Additionally, non-spatial features such as the shape and colors of perisaccadic stimuli have also been investigated to evaluate the effect of perisaccadic compression. Specifically, the discrimination of shape (Matsumiya and Uchikawa, 2001) and colors (Lappe et al., 2006; Wittenberg et al., 2008) of visual stimuli is preserved, but they are not perceived in separate positions. Although the mechanism of this effect is still an open question, the general view describes the perisaccadic mislocalization as being related to mechanisms aimed at maintaining visual stability (Matin and Pearce, 1965; Honda, 1989; Schlag and Schlag-Rey, 1995; Ross et al., 1997; Lappe et al., 2000; Pola, 2004; Binda and Morrone, 2018).

### The hand domain

**Figure 6:**
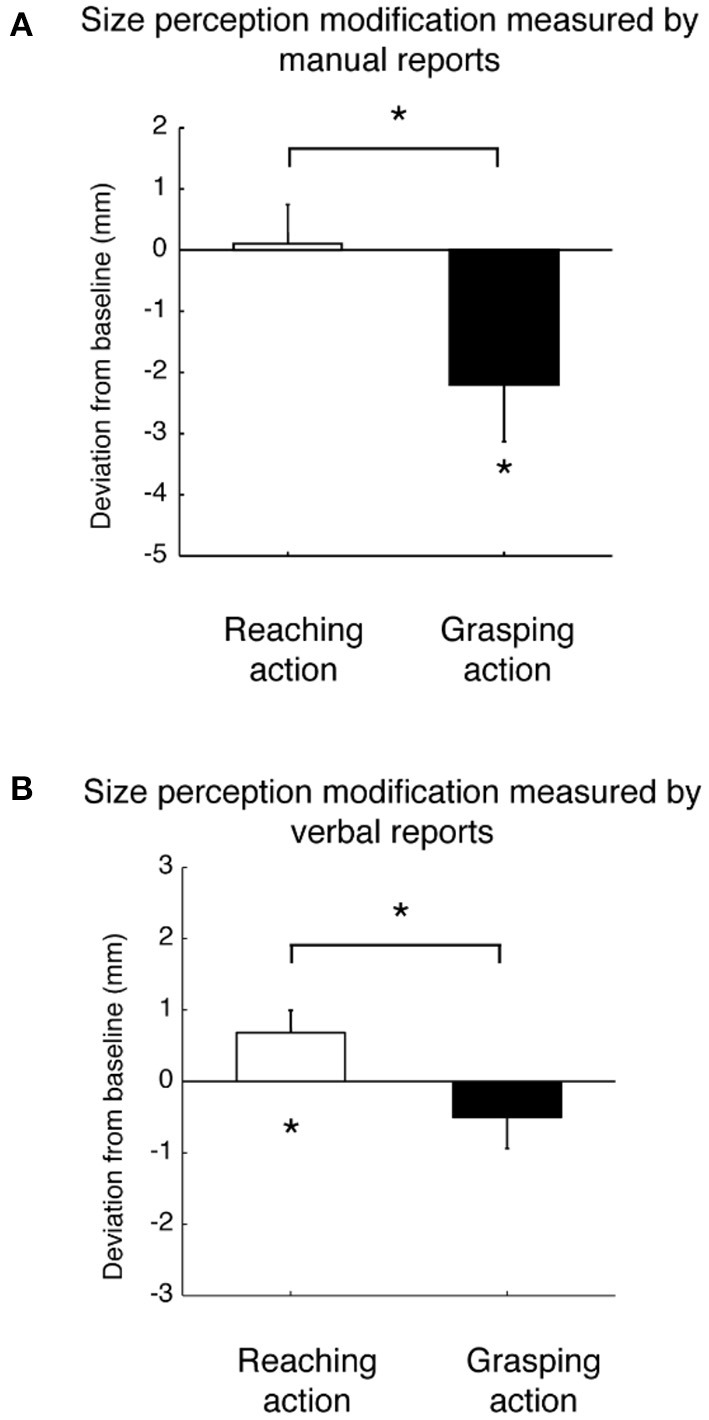
Size perception modification after reaching and grasping actions. **(A)** Mean deviation of perceptual responses by grip aperture for reaching (white column) and grasping (black column). **(B)** Mean deviation of verbal perceptual reports for reaching (white column) and grasping (black column). All data are averaged across participants and sizes. Error bars are standard errors of the mean. *p < 0.05, significance level (modified from Bosco et al., 2017).

**Figure 7:**
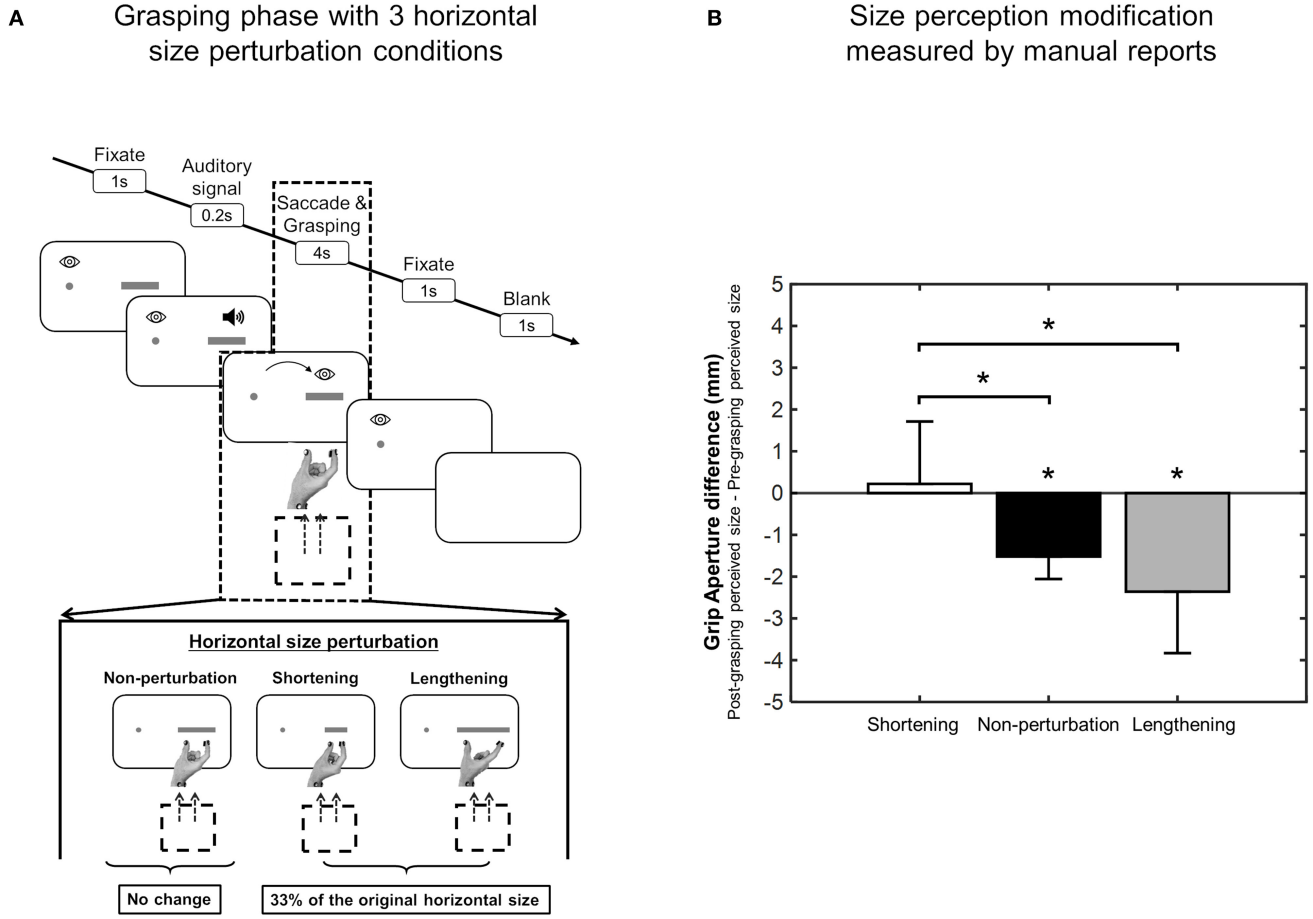
Size perception modification after size-perturbed grasping execution. **(A)** Experimental task sequence. **(B)** Mean Deviation values of grip aperture from the baseline averaged across size perturbation condition. Details as in Figure 5 (modified from Sanz Diez et al., 2022). ^*^p < 0.05, significance level.

The execution of different types of hand movements can generate perceptual modifications of object properties relevant for that type of action, such as the perception of size and weight. In 2017, Bosco et al. (2017) investigated the direct effect of reaching and grasping execution on the size perception of a visual target. They found that the change in size perception was larger after a grasping action than after a reaching action and all participants perceived objects to be smaller after the grasping compared to the reaching. These results were consistent in both manual and verbal reports, as is shown in Figure 6 (Bosco et al., 2017). Sanz Diez et al. (2022) evaluated size perception after a grasping movement performed toward a visual target that changed in size during the execution of the movement. Although the perceptual phase before and after grasping execution applied to the same target that, in these two moments of the task, was identical in size, they found that, after the grasping action, reports regarding perceptual size showed significant differences that depended on the type of size change that occurred during movement execution. In fact, as shown in Figure 7, observers reported a smaller size perception when the visual target was lengthened during the grasping execution and no perception modification when the visual target was shortened during the grasping execution (Sanz Diez et al., 2022). In both of the studies described above, the perceptual modification occurred according to the type of movement (i.e., reaching or grasping) and to the unpredictable changes of target size during the movement itself, suggesting that this modification can be considered to be a descriptive parameter of the previous motor action (Bosco et al., 2017; Sanz Diez et al., 2022).

An advantage of the effect of action execution on perception is represented by changes to the motor system obtained with skill learning. The formation and retrieval of sensorimotor memories acquired from previous hand-object interactions are fundamental for dexterous object manipulation learning (Westling and Johansson, 1984; Johansson and Westling, 1988). This allows the modulation of digit forces in a fashion that is anticipatory, i.e., before the lifting of the object (Gordon et al., 1993; Burstedt et al., 1999; Salimi et al., 2000). In a task requiring participants to lift an object while minimizing the roll caused by asymmetric mass distribution of an external torque, the implicit learning after action execution led to minimization of object roll by a re-arrangement of digit positions (Lukos et al., 2007, 2008) and a modulation of the force distribution exerted by the fingers (Salimi et al., 2000; Fu et al., 2010).

Within this perspective, it is also useful to describe the size-weight illusion (SWI), for the first time described by Charpentier (Charpentier, 1891). In fact, the SWI is visible when a subject lifts two objects of different size, but of equal weight, and reports the smaller object as being heavier. The SWI illusion is robust (Murray et al., 1999; Flanagan and Beltzner, 2000; Kawai, 2002a,b, 2003a,b; Grandy and Westwood, 2006; Dijker, 2008; Flanagan et al., 2008; Chouinard et al., 2009), and the effect is still present when the lifter knows that both objects are of the same weight (Flanagan and Beltzner, 2000). The SWI illusion has been thoroughly studied to understand the mechanism of signal integration for weight perception, and it is an example of how the sensorimotor system works in a Bayesian manner. According to this view, the nervous system combines prior knowledge regarding object properties learned by previous experience (“the prior”) with current sensory information (“the likelihood”), to appropriately estimate object property (“the posterior”) for action and perception functions (van Beers et al., 2002; Körding and Wolpert, 2006). In most cases, the combination of prior and likelihood generates correct perception and behavior, but perception can be misleading. In the case of SWI, for example, the prior is perceived higher than the likelihood, generating a perception that does not correspond with the actual physical properties of the object. However, the repetition of the lifting action recalibrates the perception of weight, and the force distribution is adjusted according to the real weight of the objects. Although there is still no consensus as to the process that gives rise to the SWI, an objective aspect is that the execution of the manipulation action on the objects has a pragmatic effect on weight and size perception.

### The leg domain

Walking interaction leads to a perception-action recalibration, and it is typically investigated by the measurement of perceived size or perceived distance. This is because, according to the size-distance invariance hypothesis (Sedgwick, 1986), size and distance perception are strictly coupled. Brenner and van Damme (1999) found that perceived object size, shape, and distance are largely independent. Although object size, shape, and distance estimations were similarly affected by changes in object distance perception, modifications in perceived shape caused by motion parallax did not affect perceived size or distance. This indicates their independence. Although the direct relationship between size and distance perception has been debated in this study, the judgements of distance and size have been shown to be tightly linked in other studies (Gogel et al., 1985; Hutchison and Loomis, 2006). Results showing an improvement in judgments of distances after the walking interaction were found by Waller & Richardson (Waller and Richardson, 2008). The same authors showed that distance judgments in a virtual environment were unaffected by interactions in which participants viewed only a simulation of visual walking (i.e., optic flow only). This suggested that a body-based movement is necessary. Furthermore, perceptual reports of distance increased in accuracy after participants performed a blind-walking task consisting in the receipt of visual or verbal feedback (Richardson and Waller, 2005; Mohler et al., 2006). The results showed the sufficiency of body-based interaction. Kelly et al. (2013) found that perceptual reports of object size improved after a walking interaction, because an increase in perceived distance was observed. The finding that perceptual reports regarding size improved after the interaction indicates that walking leads to a rescaling of space perception and not only to a simple recalibration of walked distance (Siegel et al., 2017). In open-loop blind walking tasks, calibration and recalibration of locomotion has been observed. In these tasks, an observer views a target on the ground and, after closing his/her eyes, he/she has to walk toward the target without seeing. In normal conditions, blind walking performance is quite accurate and reflects the perception of the target location (Rieser et al., 1990; Loomis and Philbeck, 2008). After manipulation of the rate of environmental optic flow in relation to the biomechanical rate of normal walking, observers undershot the target when the environmental flow was faster, and overshot the target when environmental flow was slower compared to the perception of normal walking speed (Rieser et al., 1995). Additionally, studies investigating the visual perception of egocentric distances showed that perceptual judgments (e.g., verbal reports) showed a systematic underestimation of egocentric distances (Foley, 1977; Li and Giudice, 2013), while blindfolded walking toward a remembered target location was executed more accurately (Loomis et al., 1992; Li and Giudice, 2013). Although the former suggests that a systematic compression of physical distance is visually perceived, visually directed walking is not affected by this perceptual distortion.

## Conclusions and future perspectives

**Figure 8:**
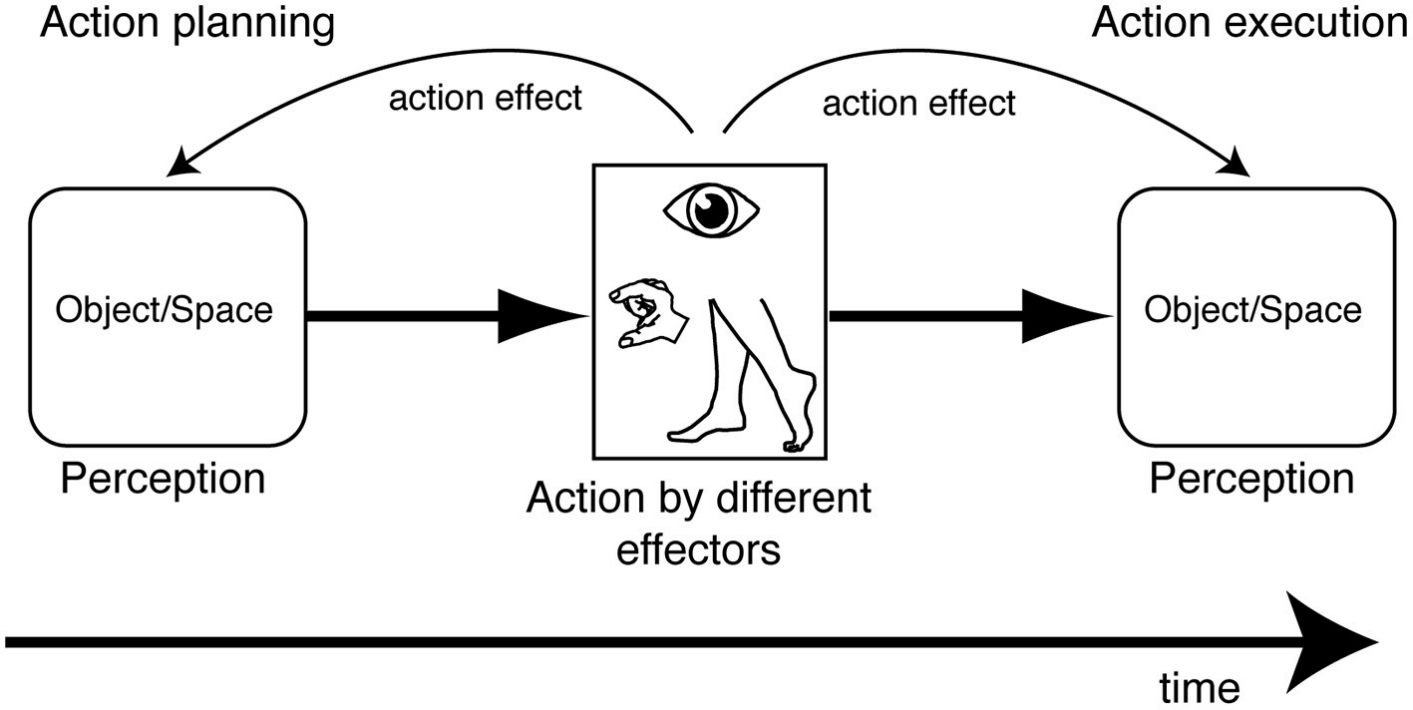
Summary of object/space perception modulation by action planning and action execution evoked by movement of different effectors.

A multitude of works have been presented showing the effect of actions performed with the eyes, the hands, and the legs on visual perception of objects and space using different approaches and paradigms. The action influence is present before and after execution of the movement, suggesting that visual perception, when it is integrated with the action, is “ready to act” (before execution) and is transformed by action execution (see Figure 8 for a summary). In both cases, the perceptual responses, collected in different ways, are parameters that describe the subsequent or previous motor responses. This suggests a mechanism which exchanges information between the motor and perceptual system when we are in a specific visuomotor contingency. At a behavioral level, we can take advantage of these aspects because they can be used as action intention predictors when they occur during action planning and, interestingly, as a postdictive component that specifies the previous motor experience when they occur after action execution. In this latter case, the postdictive perceptual component also updates the information that is necessary for a potential subsequent action. The use of the action-based perceptual information can be helpful in all those artificial intelligent (AI) systems that are used with motor assistive devices. In fact, the use of perceptual information during action planning can be implemented with other parameters (e.g., neural signals) to extract action intentions that exploit the residual motor abilities of different effectors that are necessary to give perceptual responses by pressing a button, for example, or extending only certain fingers and not others. The use of perceptual information after action execution can be implemented in AI systems that are able to communicate with humans, with the objective of creating a mutual learning exchange. In fact, the modification of perception following the execution of a particular movement may be used as a feedback signal, in order to correct a subsequent motor response and compensate for the error due to previous AI action decisions. This allows the system to improve the outcome of the action and, consequently, increases the user's trust in the AI system.

## Author contributions

AB: conceptualization, visualization, writing original draft, and writing—review and editing. SDP: visualization and writing original draft. MF: writing—review and editing. PF: writing—review and editing and funding acquisition. All authors contributed to the article and approved the submitted version.
